# Microbial quality of edible seeds commercially available in southern Portugal

**DOI:** 10.3934/microbiol.2022004

**Published:** 2022-02-22

**Authors:** Daniela Silva, Patrícia Nunes, Jessie Melo, Célia Quintas

**Affiliations:** 1 Universidade do Algarve, Instituto Superior de Engenharia, Campus da Penha,8005-139, Faro Portugal; 2 MED, Mediterranean Institute for Agriculture, Environment and Development, Universidade do Algarve, Campus de Gambelas, 8005-139 Faro, Portugal

**Keywords:** Microbial quality, edible seeds, sesame, flaxseed, chia, pumpkin, sunflower

## Abstract

In the present work, the microbiological quality of sesame, flaxseed, chia, pumpkin sunflower seeds, a mix of seeds, as well as flaxseed flour, marketed in southern Portugal, were studied through the counting of aerobic microorganisms at 30 °C (AM), molds and yeast (M&Y), *Escherichia coli* (β-glucuronidase positive) (β-GP *E. coli*), *Staphylococcus* coagulase positive, and detection of *Salmonella* spp. The persistence of AM and M&Y populations were also counted in organic and non-organic flaxseed at 20 °C for 11 months. The seeds with the highest average of AM were flaxseed (1.3 x 10^6^ CFU/g) followed by flaxseed flour (1.1 x 10^6^ CFU/g) while the lowest level was found in chia (2.9 x 10^4^ CFU/g). This seed also presented the lowest average values of filamentous fungi (9.8 x 10^2^ CFU/g), whereas sunflower seeds had the highest levels (1.7 x 10^5^ CFU/g). Flaxseed flour had the highest yeast counts (1.5 x 10^4^ CFU/g). Although some samples had high levels of AM and fungi, β-GP *E. coli* and *Salmonella* were not detected, therefore, they complied with the microbiological criteria of the European Union. The organic flaxseed contained higher numbers of AM and M&Y than the non-organic ones (p < 0.05). In addition, the storage of flaxseed at 20 °C resulted in changes of AM and M&Y, showing that these populations were able to remain viable after eleven months (AM Log 5.4–Log 5.6; M&Y Log 2.8–Log 4.1). The results obtained in the present study, namely those high levels of AM and fungi (>10^6^ and 10^4^ CFU/g respectively), alert to the need of improving processing practices, storage/distribution conditions of edible seeds and derivatives, as well as the requirement of implementing adequate decontamination techniques.

## 1. Introduction

Edible seeds bring color and flavor, in addition to essential nutrients to a huge variety of dishes, which can include snacks, drinks, breakfast, salads, desserts, among others [1]. Nutritionally, they are an excellent source of protein, fiber and fat, and are among the richest vegetable sources in α-linoleic acid, being approximately 60% of the total fatty acid content of seeds [2]. Some authors have also measured high levels of antidiabetic and antioxidant agents in seeds [3]. Several edible seeds are consumed mainly because of their nutritional, phytochemical and therapeutic potential. Flaxseed (*Linum usitatissimum*) is a good source of lignans, which appear to have antioxidant properties [4]. Pumpkin seeds (*Curcubita* sp.) are also associated to health benefits specially in preventing prostate cancer and as potential functional foods [5],[6]. Other seeds, such as sesame (*Sesamum indicum*), are rich in oil (50%) and contain minor nutrients in high levels, for example, iron and calcium [7]. Dietary fibers and high amounts of phytochemicals are present in chia seeds (*Salvia hispanica*) which could mean a benefit to consumers health and its usage in food industries has increased [8]. The sunflower (*Helianthus annus*) seeds are rich in folic acid, which is important for pregnant women's cellular metabolism [9].

These food products are grown, harvested, cleaned and mechanically sorted in a similar way to cereals. In some situations, they are subjected to drying processes, such as sunflower seeds [10]. Therefore, microbial colonization in edible seeds is affected by the practices and conditions that occur during pre-harvest, harvest and post-harvest processing. During the pre-harvest, growing plants are susceptible to a wide range of microbial contamination sources, for example: the soil, animal excrement and irrigation water quality. Harvesting includes several stages, such as collecting, classification, packaging and transportation, and in these stages contaminating microbial agents can be introduced through cross-contamination from equipment, containers, transport vehicles and workers. In the post-harvest, possible sources of contamination are materials and equipment, lack of hygiene and even handlers and environments. Size reduction operations, such as in the production of flours, are also responsible for increasing microbial contamination in seed derivatives, when subjected to milling or other size reduction operations. Although seeds are low water activity (a_W_) foods and generally considered microbiologically safe [11],[12], various outbreaks have been reported in different parts of the world. In 2002, outbreaks caused by *Salmonella* were identified in Australia and New Zealand, associated with the consumption of imported tahini (sesame paste) [13]. Between 2007 and 2008, a study was carried out in the United Kingdom to assess the microbiological safety of edible seeds through the detection of *Salmonella* spp. and enumeration of *Escherichia coli*. In this study, which involved 3,735 seed samples, *Salmonella* spp. was detected in 0.6% of the samples, of which 57% were sesame seeds. The rest of the contaminated samples were seed mixtures, sunflower seeds, melon seeds and flaxseed. In the same study, *E. coli* was detected in 9% of the samples, with 1.5% containing unsatisfactory values (≥10^2^ CFU/g) [10]. However, higher values of *Salmonella* prevail in sesame (12%) and chia (31%) were detected by Arana et al [14]. In 2014, a chia seed meal was associated with an outbreak of *Salmonella*, which involved 63 cases recorded in Canada and 31 in the United States [15]. In addition, in the United States, several edible seeds, such as chia, sunflower and pumpkin seeds, have been subjected to multiple recalls from the market due to the presence of *Salmonella* [15]. The same authors alerted to *Salmonella* outbreaks caused by contaminated sprouts derived from previously contaminated dried seeds. The presence of *Salmonella* in seeds is worrying as it has been shown that low a_W_ values and high lipid concentrations seem to have a protective effect on *Salmonella* [16]. In addition, Nascimento et al [17] alert to the fact that high fat concentrations protect these bacteria from gastric acidity. Furthermore, edible seeds are usually eaten raw or minimally processed as they are added to snacks/dishes which are consumed without any heat treatment. Considering the increasing consumption of edible seeds, the recorded outbreaks in recent years, along with the seeds withdrawal from the market in some countries, it is relevant to assess the microbiological quality of these foods. Moreover, there are no legal regulations or guidelines in the European Union that include microbiological criteria for edible seeds, although the European Commission [18],[19] imposes that food commodities should not contain pathogenic bacteria which represent public health risks.

The present study intended to investigate the microbial quality of edible seeds and flaxseed flour available in the Portuguese commerce. A comparison of the microbial contamination of flaxseed obtained from organic and non-organic farming and the evaluation of the microbial populations during eleven months was also performed.

## 2. Materials and methods

### 2.1. Samples

The microbiological quality of 126 packed edible dried (not cooked) seeds and flaxseed flour acquired from commercial places in the municipality of Faro (Algarve) was evaluated. Different types of seeds were tested: sesame (18 samples); flaxseeds (18 samples); sunflower (18 samples); pumpkin (18 samples); and chia (18 samples) as well as a seed mixture (18 samples). The microbial quality of flaxseed flour (18 samples) was also assessed. All the samples were transported to the Microbiology Laboratory of the Institute of Engineering of the University of Algarve.

### 2.2. Microbial analyses

The microbiological analyses were done following the methodologies described by the International Organization for Standardization (ISO) with adaptations. The packed edible seed samples were assessed for aerobic microorganisms count at 30 °C (AM) [20], molds and yeasts (M&Y) [21], β-glucuronidase positive *Escherichia coli* (β-GP *E. coli*) [22], and coagulase positive staphylococci [23], as well as detection of *Salmonella* spp. [24].

#### 2.2.1. Detection of *Salmonella* spp.

The methodology described in ISO 6579 [24] was carried out to detect *Salmonella* spp. The pre-enrichment step was done using 25 g (in duplicate) of each type of seeds diluted in 225 mL buffered peptone water (BPW, OXOID) and homogenized in a Stomacher (Model 400 Circulator, Seward, Norfolk, England) during three minutes. From this suspension the enumeration of *E. coli*, and staphylococci were also prepared. The remaining pre-enrichment was incubated at 37 °C for 18 h for the detection of *Salmonella* spp. After incubation, aliquots of the pre-enrichment mixture were inoculated in Rappaport-Vassiliadis Enrichment Broth (OXOID) and Muller-Kauffmann Tetrathionate-Novobiocin Broth (OXOID) and were incubated during 24 h at 41.4 °C and 37 °C, respectively. The suspensions were then plated out on the surface of two selective media, Xylose Lysine Deoxycholate Agar (SCHARLAU) and Brilliance Salmonella Agar (OXOID). After 24 h of incubation, the plates were examined for the presence of typical and non-typical colonies, which were then subjected to biochemical confirmation.

#### 2.2.2. Enumeration of *Escherichia coli*

*Escherichia coli* was enumerated according to the ISO 16649-2 [22] incorporating the aliquots of 1 mL the in Chromocult Triptone Bile X-glucuronide Agar (TBX) (MERCK) (in duplicate), following an incubation at 44 °C during 24 h. This method enumerates β-glucuronidase positive *E. coli (*β-GP *E. coli*).

#### 2.2.3. Enumeration of coagulase positive staphylococci

The enumeration of the coagulase positive staphylococci was done inoculating Baird-Parker medium (OXOID) with 0,1 mL of the dilution (in duplicate), followed by an incubation at 37 °C, during 24–48 h, according to the ISO 6888-1 (1999; Amd 1:2003) [23]. Typical and atypical colonies were tested for coagulase reaction using the *Staphylase* Test (OXOID).

#### 2.2.4. Enumeration of aerobic microorganisms at 30 °C (AM)

The counting of AM was done as described in the ISO 4833 [20]. Subsamples of ten grams of each seed package (in duplicate) were diluted in 90 mL of Ringer solution (OXOID) and homogenized in a stomacher (Model 400 Circulator, Seward, Norfolk, England) during three minutes. Inoculations were done in duplicate, incorporating aliquots (1 ml) of original mix and dilutions, in Plate Count Agar (SCHARLAU) following a 72-h incubation at 30 °C.

#### 2.2.5. Enumeration of molds and yeasts (M&Y)

The enumeration of M&Y was done following the ISO 21527-1 [21]. Subsamples of ten grams of each seed package (in duplicate) were diluted in 90 mL of Ringer solution (OXOID) and homogenized in a stomacher (Model 400 Circulator, Seward, Norfolk, England) during three minutes. Inoculations were done in duplicate, spreading the aliquots (0.1 mL) of original mix and dilutions on Dichloran Rose Bengal Chloramphenicol Agar (OXOID) following a 5–7 days incubation at 25 °C.

#### 2.2.6. Persistence of aerobic microorganisms and molds and yeasts in flaxseed (organic and non-organic) during eleven months

In addition to the study of the microbial quality of packaged seeds described previously, the microbial quality of flaxseed sold in bulk resulting from organic and non-organic farming was done throughout a storage period of 11 months, at 20 °C. The seeds were divided in polyethylene packages (90 µm) containing 12 g each. The bags were closed with a soft vacuum (4 out of 10) and medium sealing (4 out of 10) using a packaging and sealing machine (Quick Pack, Speedy, Italy), and left at a constant temperature of 20 °C during 11 months. 20 bags of each type of seed were prepared. In the beginning of the study the flaxseeds were checked for *Salmonella* spp., β-GP *E. coli*, staphylococci as described previously, as well as for aerobic microorganisms at 30 °C (AM) and filamentous fungi and yeasts (M&Y). During the eleven-month storage, the seeds were only analyzed for the counting of AM and M&Y. In the first five months, the seeds were analyzed once a month and then three more times until the eleventh month. At every moment of analysis two bags of 12 g were studied for both type of flaxseed. The bulk seeds were purchased from local supermarkets and transported to the Microbiology Laboratory of the Institute of Engineering of the University of Algarve.

### 2.3. Data analysis

Duplicates of the analyses were done for all samples. The minimum, maximum and average values of colony forming units (CFU/g) of each type of seed were recorded. Regarding the organic and non-organic flaxseed, the results were presented as the average ± standard deviation (log CFU/g). Statistically significant differences among samples were tested using a post-hoc comparison test (Tukey's test) at α = 0.05. The differences of AM and M&Y on flaxseed obtained from organic and non-organic throughout the time were assessed by factorial ANOVA. Differences were considered statistically significant at *p* < 0.05. Statistics were carried out by IBM SPSS software version 27.0 (SPSS, Inc., Chicago, IL).

## 3. Results and discussion

In none of the tested samples (50 g) β-glucuronidase positive *Escherichia coli* (β-GP *E. coli*), coagulase positive staphylococci and *Salmonella* spp. were found, contrary to the results of Willis et al. [10] and Arana et al. [14] where some seed samples were contaminated with *Salmonella* and *E. coli*. However, in the present work the tested seeds revealed high levels of total microbiota including aerobic microorganisms counted at 30 °C (AM) and fungi (M&Y).

**Table 1.** Aerobic Microorganisms counted at 30 °C (AM) (CFU/g) of edible seeds and derivatives (N-Number of samples of each product; ^a^Microbial population in CFU/g of seed).

Seed samples	*N*	<10^1^	10^1^–10^2^	10^2^–10^3^	10^3^–10^4^	>10^4^	Minimum^a^	Maximum^a^	Average^a^
Sesame	18	1	1	3	2	11	<1.0 x 10	8.0 x 10^6^	6.0 x 10^5^
Flaxeed	18			1	4	13	7.9 x 10^2^	9.4 x 10^6^	1.3 x 10^6^
Pumpkin	18				4	14	3.6 x 10^2^	6.8 x 10^5^	1.2 x 10^5^
Sunflower	18		1	6	10	1	1.0 x 10^2^	6.9 x 10^5^	4.0 x 10^4^
Chia	18		1	11	3	3	8.0 x 10^1^	2.3 x 10^5^	2.9 x 10^4^
Mix of seeds	18		1	1	3	13	2.5 x 10^1^	2.5 x 10^6^	2.8 x 10^5^
Flaxseed flour	18				1	17	4.3 x 10^3^	4.1 x 10^6^	1.1 x 10^6^

Total	126	1	4	22	27	72	<1.0 x 10	9.4 x 10^6^	5.0 x 10^5^

The AM ranged from values below 1.0 x 10 to 9.4 x 10^6^ CFU/g (Table 1). About 42.86% of the samples studied, had values lower than or equal to 10^4^ CFU/g. The seeds that presented a lower average level of this type of microbiota were the chia seeds (2.9 x 10^4^ CFU/g). Those with the highest average level were flaxseeds (1.3 x 10^6^ CFU/g), followed by flaxseed flour (1.1 x 10^6^ CFU/g). In the study of Shah et al. [25] values between 10^4^ and 10^5^ CFU/g of AM for flaxseed and corresponding flour, respectively, were reported. These values are lower than those obtained in the present work. The results of Al-Bachir et al. [26], for sesame seeds, are also inferior to those obtained in the present study. The aforementioned author reported 3.16 Log CFU/g, while in the current work an average AM value of 5.79 Log CFU/g (6.0 x 10^5^ CFU/g) was recorded.

With regard to the counts of filamentous fungi, these microorganisms ranged between the detection limit (1.0 x 10) and 3.1x10^6^ CFU/g (Table 2). About 27.78% of the samples studied, contained values less than or equal to 10^2^ CFU/g. The seeds that presented a lower average level of this type of microbiota were the chia seeds (9.8 x 10^2^ CFU/g). Those with the highest average level were sunflower seeds (1.7 x 10^5^ CFU/g), followed by pumpkin seeds (9.8 x 10^3^ CFU/g). Flaxseed flour was the product with the highest number of samples containing high numbers of filamentous fungi (>10^3^ CFU/g), followed by flaxseed, sunflower and sesame seeds. Robertson et al. [27] enumerated filamentous fungi in sunflower seeds, having reported values of 4.11 Log CFU/g, lower than the average of counts enumerated in the samples analysed in the current work. Regarding the counts of filamentous fungi in sesame seeds, Al-Bachir [26] reported 2.28 Log CFU/g, a value lower than the average found in the present study (3.3 x 10^3^ CFU/g).

**Table 2.** Filamentous fungi (CFU/g) in seeds and derivatives (N-Number of samples of each product; ^a^Microbial population in CFU/g of seed).

Seed samples	*N*	<10^1^	10^1^–10^2^	10^2^–10^3^	10^3^–10^4^	>10^4^	Minimum^a^	Maximum^a^	Average^a^
Sesame	18	6		5	5	2	<1.0 x 10	1.4 x 10^4^	3.3 x 10^3^
Flaxseed	18	3	1	7	7		<1.0 x 10	8.5 x 10^3^	1.7 x 10^3^
Pumpkin	18	5	3	7	1	2	<1.0 x 10	1.5 x 10^5^	9.8 x 10^3^
Sunfllour	18	1	3	6	5	3	<1.0 x 10	3.1 x 10^6^	1.7 x 10^5^
Chia	18	5	3	7	2	1	<1.0 x 10	1.1 x 10^4^	9.8 x 10^2^
Mix of seeds	18	2		12	3	1	<1.0 x 10	2.0 x 10^4^	1.8 x 10^3^
Flaxseed flour	18	3		4	10	1	<1.0 x 10	1.4 x 10^4^	2.3 x 10^3^

Total	126	25	10	48	33	10	<1.0 x 10	3.1 x 10^6^	2.8 x 10^4^

The yeast counts in the seeds varied between the detection limit (1.0 x 10) and 9.6 x 10^6^ CFU/g (Table 3). About 58.73% of the samples studied contained values less than or equal to 10^2^ CFU/g. The seeds that presented a lower average level of this type of microbiota were sunflower seeds (1.4 x 10 CFU/g). The samples with the highest average level were flaxseed flour (1.5 x 10^4^ CFU/g), followed by flaxseed (7.3 x 10^3^ CFU/g). High levels of yeasts may result in product flavor change and also cause spoilage [28].

According to the Portuguese guidelines, “Guide values for the evaluation/interpretation of the microbiological quality of ready-to-eat foods and surfaces of food preparation as well as distribution environment” [28], the existence of a large number of AM does not mean that the food product is contaminated with pathogenic microorganisms or spoiled but it may suggest that good practices were not followed during production, processing or storage. AM content may be high in case of non-compliance with good manufacturing practices, use of low-quality raw materials, deficient environment sanitation conditions, occurrence of cross contaminations, failure/ insufficient treatments and prolonged stay in inadequate conditions during shelf-life and distribution. In addition, the AM is also an adequate parameter to assess the potential for deterioration of the food/ingredient. In fact, levels higher than 10^6^ CFU/g were found in 2, 3, 2 and 8 samples of sesame, flaxseed, mix of seeds and flaxseed flour, respectively, which indicate a high risk of degradation [28]. According to the guidelines from Luxembourg (Critéres microbiologiques des déenres alimentaires, Luxembourg) [29] values of filamentous fungi higher than 10^4^ CFU/g (established for nuts and nut products and snack products) are associated with spoilage. In a number of samples of edible seeds studied, the filamentous fungi enumerated were higher than 10^4^ CFU/g, namely in 2 samples of sesame, 2 of pumpkin, 3 of sunflower, 1 of chia, 1 of seed mixtures and 1 of flaxseed flour. In fact, one of the possible hazards with edible seeds is the high contamination by mycotoxins, resulting from the presence of certain species of filamentous fungi. Arroyo-Manzanares et al. [30] detected sunflower seeds contaminated with several mycotoxins and in the present work, 8 samples of sunflower seeds contained high levels of filamentous fungi.

**Table 3.** Yeasts (CFU/g) in seeds and derivatives (N-Number of samples of each product; ^a^Microbial population in CFU/g of seed).

Seed samples	*N*	<10^1^	10^1^–10^2^	10^2^–10^3^	10^3^–10^4^	>10^4^	Minimum^a^	Maximum^a^	Average^a^
Sesame	18	14		2	2		<1.0 x 10	1.0 x 10^4^	6.7 x 10^2^
Flaxseed	18	1	2	4	6	5	<1.0 x 10	3.2 x 10^4^	7.3 x 10^3^
Pumpkin	18	7	3	3	3	2	<1.0 x 10	8.5 x 10^4^	6.3 x 10^3^
Sunflower	18	16	1	1			<1.0 x 10	1.5 x 10^2^	1.4 x 10^1^
Chia	18	12		3	3		<1.0 x 10	8.5 x 10^3^	9.0 x 10^2^
Mix of seeds	18	9	3	3	2	1	<1.0 x 10	2.4 x 10^4^	1.6 x 10^3^
Flaxseed flour	18	6		2	6	4	<1.0 x 10	9.6 x 10^4^	1.5 x 10^4^

Total	126	65	9	18	22	12	<1.0 x 10	9.6 x 10^4^	4.6 x 10^3^

**Figure 1.**
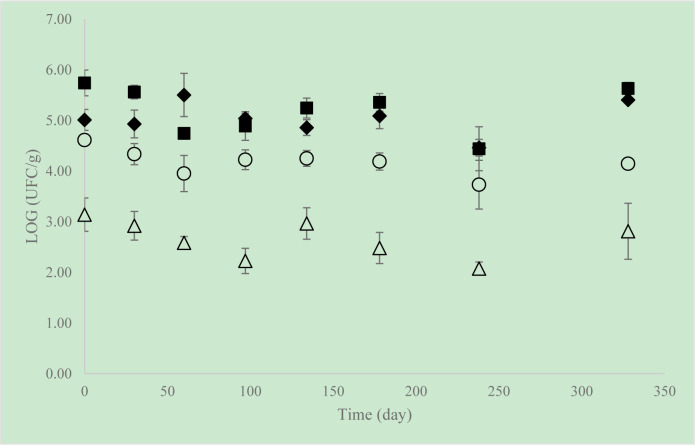
Microbiological quality of biological flaxseeds and non-biological flaxseeds, during almost 11 months, at 20 °C (█ AM organic flaxseeds; ♦ AM non-organic flaxseeds; ○ M&Y organic flaxseeds; Δ M&Y non-organic flaxseeds) (Results are presented as the average ± standard deviation (log CFU/g)).

In a second part of this work, the levels of AM and molds and yeasts (M&Y) in flaxseeds from organic and non-organic farming, available on the market, were compared. The variation of these populations in seeds conditioned at 20 °C for approximately 11 months was also studied (Figure 1). Initially, the biological seeds had an average AM population of 5.74 Log CFU/g and M&Y of 4.61 Log CFU/g, while the non-biological flaxseed had average levels of AM of 5.01 Log CFU/g and of M&Y of 3.14 Log CFU/g, being these differences statistically significant (p < 0.05). The AM population showed slight variations over time in the two types of samples. At the end of the storage period, the AM were recorded at average levels of 5.63 Log CFU/g in biological seeds, which was not significantly different from the value counted in non-biological seeds, 5.41 Log CFU/g (p > 0.05). No significant differences were detected in the levels of AM in the two types of flaxseed after day 97. The differences in the counting of M&Y in both type of seeds were significant (p < 0.05) during the storage period where the non-biological seeds presented levels of fungal contamination lower than those of the biological seeds, with average values of 2.81 Log CFU/g and 4.14 Log CFU/g, respectively, being recorded at the end of the study. According to Shah et al. [25] the AM population remained practically unchanged over a 36-week storage period. Edible seeds are foods of low a_W_ (<0,70) [11] yet the AM and M&Y, in the present study, were able to remain viable for 11 months. In addition, Arana et al [14] found that *Salmonella* survived at least 240 days in chia and sesame seeds. Although edible seeds are low a_W_ foods and perceived as microbiologically safe, there are some major concerns related to them. One of these concerns is the survival of some microorganisms, including spoilers and pathogenic, remaining viable for long periods of time (various months and even years). In addition, when these seeds are used as ingredients in the preparation of high a_W_ foods, the microbial growth may be favored, leading to a faster degradation or being responsible for consumer diseases [11],[12].

The seeds studied in this work were purchased from several commercial brands, from several countries, produced in different climatic and environmental conditions, so the evaluation of microbiological quality results is related to intrinsic factors, but also to different production and processing conditions in various regions of the world. Seeds are exposed to a wide variety of contaminants, including fungi. This problem becomes a relevant issue in some countries where the characteristics of the climate, agricultural practices and storage conditions are favorable to fungal proliferation and consequently to toxin production [31].

The results obtained in this study highlight the importance of improving manufacturing and hygiene practices at all levels of the production/processing chain in order to prevent and reduce microbial contamination of the final product. Critical control points should be identified throughout the various production/processing operations of edible seeds. These seeds are grown in extensive agriculture areas and collected, cleaned and sorted in similar ways to cereals. Willis et al. [10] call attention to the drying operation as a critical step which should include an adequate heat treatment to reduce unwanted microbiota. In most cases the drying process relies on the seeds distribution on the ground and left to dry in the sun. They may also be dried through the application of hot air in mechanically agitated equipment. Other authors [32] report that the roasting of seeds, an operation applied in some situations, contributes to the control of microbial contamination in this type of food. However, very high temperatures can negatively affect the nutritional quality of edible seeds. In all situations where decontamination treatments are applied, measures must be taken to avoid subsequent cross-contamination.

Currently edible seeds are commercialized within global systems, where the source of cultivation and harvesting are distant from points of sale and these products may reach the consumers through a long and complex food chain. Therefore, food safety of edible seeds became a relevant public health issue in the current food market.

Good agricultural practices should be implemented, coupled with good manufacturing practices by the food industry. Improved drying, storage and package conditions, along with appropriate decontamination techniques and good hygienic practices, such as equipment disinfection and hygiene control protocols are crucial guidelines to minimize the risk of cross contamination. This study highlights the potential hazard associated with the consumption of edible seeds and calls attention to the need of safer hygiene measures through all production stages in order to lower the microbial contamination reducing food degradation and public health threats.

## 4. Conclusion

The present work gives an overview of the microbiological quality of edible seeds commercialized in Southern Portugal markets. The high number of filamentous fungi found in some samples warns to the potential of these foods as being vehicles for mycotoxins, representing a health hazard for consumers. The existence of microbial populations at high levels in a large number of samples, as well as their ability to remain viable, shows the importance of increasing microbiological control at certain steps of their production/processing/storage process. On the other hand, the high loads of fungi and yeasts on the organic flaxseed samples, compared to the non-organic ones reinforces the attention to the importance of an appropriate antimicrobial treatment, applied to the seeds to ensure a reduction on fungal contamination. It can also address the need for good hygiene measures during all stages of the chain, including food distribution and sale. Furthermore, training programs for farmers, manufacturers and traders should be encouraged by food safety authorities as part of an integrated strategy to improve the general microbiological quality of edible seeds.
